# Lightweight colon polyp segmentation algorithm based on improved DeepLabV3+

**DOI:** 10.7150/jca.88684

**Published:** 2024-01-01

**Authors:** Shiyu Xiang, Lisheng Wei, Kaifeng Hu

**Affiliations:** 1 School of Electrical EngineeringAnhui Polytechnic University, Wuhu 241000, China.; 2 Anhui Key Laboratory of Electric Drive and Control, Wuhu 241000, China.; 3 The First Affiliated Hospital of Wannan Medical College Wuhu, Wuhu 241001, China.

**Keywords:** DeepLabV3+, Image segmentation, Feature fusion, Transfer learning

## Abstract

To address the problems that the current polyp segmentation model is complicated and the segmentation accuracy needs to be further improved, a lightweight polyp segmentation network model Li-DeepLabV3+ is proposed. Firstly, the optimized MobileNetV2 network is used as the backbone network to reduce the model complexity. Secondly, an improved simple pyramid pooling module is used to replace the original Atrous Spatial Pyramid Pooling structure, which improves the model training efficiency of the model while reducing the model parameters. Finally, to enhance the feature representation, in the feature fusion module, the low-level feature and the high-level feature are fused using the improved Unified Attention Fusion Module, which applies both channel and spatial attention to enrich the fused features, thus obtaining more boundary information. The model was combined with transfer learning for training and validation on the CVC-ClinicDB and Kvasir SEG datasets, and the generalization of the model was verified across the datasets. The experiment results show that the Li-DeepLabV3+ model has superior advantages in segmentation accuracy and segmentation speed, and has certain generalization abilities.

## Introduction

Colorectal cancer is one of the cancers with the highest mortality in clinics. It has a long process of lesions, initially benign polyps caused by colon polyps, but if the examination and treatment are not timely, the benign polyps will gradually develop into pre-cancerous lesions with the development of time 1. Therefore, early screening and diagnosis of colon polyps are considered to be an effective means to prevent the occurrence of Colon adenocarcinoma. As traditional polyp detection mainly relies on the manual operation of clinicians, working for a long time can easily lead to miss detection and misdetection, the auxiliary diagnostic means combined with artificial intelligence has been widely noticed and researched 2.

Relevant experts and scholars have conducted a lot of research on polyp segmentation and have achieved rich results. Ganz et al. proposed a new method called Shape-UCM, which is an extension of the gPb-OWT-UCM algorithm, a state-of-the-art algorithm for boundary detection and segmentation, Shape-UCM can segment polyp regions, but the accuracy needs to be improved 3. Bernal et al. to solve the problem of mislocation, the energy map of window median depth of valleys accumulation was used to detect intestinal polyps and to obtain the polyp boundary 4. Although the above methods can achieve simple segmentation of polyps, they only consider part of the feature information, and features such as the texture and shape of the polyp and background information are not considered comprehensively, resulting in segmentation results with rough edges, voids, and other problems. With the development of deep learning technology, the colon polyp segmentation algorithm based on deep learning has been deeply studied 5-8. Tomar et al. proposed a text-guided attention architecture (TGANet) to solve the problem of variable size and the number of polyps, to achieve robust polyp segmentation 9. To solve the challenge in the polyp segmentation task, Sharma et al. proposed the Li-SegPNet model, which uses pyramid pooling of space to deal with the problem of segmenting objects on multiple scales and also solved the semantic gap between encoder and decoder by using attention-gated modified skip connection 10. Ige et al. proposed a Context Feature Refinement (CFR) module to solve the challenges of model generalization and low segmentation performance. By using multiple parallel convolution layers to extract context information from the incoming feature map and gradually increasing the kernel size, the network can effectively identify and segment fine details in the input image 11. To address the challenge of having no obvious boundary between polyps and their surroundings, Zhou et al. proposed a cross-level feature aggregation network (CFA-Net) for polyp segmentation, which consists of a boundary prediction network and a polyp segmentation network. The boundary prediction network is used to generate boundary-aware features which are merged into the polyp segmentation network in a layer-by-layer strategy, and a two-stream structure is used to capture hierarchical semantic information in the polyp segmentation network 12. Shen et al. proposed a dual-encoder image segmentation network including HarDNet68 and Transformer branch, meanwhile, an adaptive fusion module is used to realize the inter-channel information interaction, which in turn improves the accuracy of the segmentation network 13. Considering the clinical need for lightweight polyp segmentation models, some experts have taken the lightweight feature into account in the design of polyp segmentation models. Jeong et al. adjusted the hyperparameters based on the DeepLabV3+ model of the MobileNetV3 encoder, and compared the quantitative and qualitative results, selecting the model that performed well in terms of accuracy and speed 14. Mahmud et al. proposed PolypSegNet, a polyp segmentation model based on the Unet architecture. To reduce the major architectural constraints of the traditional Unet structure, three major building blocks were added to the baseline Unet structure, which are the sequential Depth Dilated Inception (DDI), Depth Fusion Skip Module (DFSM), and Depth Reconstruction Module (DRM). The results show that the improved model reduces the number of network parameters and memory requirements while providing accurate segmentation results 15.

Based on the above literature, although the performance of the polyp segmentation algorithm has been enhanced from different aspects, and achieved certain positive results. However, polyp segmentation has high requirements in clinical practice, so it is necessary to further improve the polyp segmentation algorithm in terms of optimization accuracy, lightweight, and generalization. In this paper, we propose a lightweight segmentation network model Li-DeepLabV3+ for polyps. Firstly, the optimized lightweight MobileNetV2 is used as the backbone network to extract image features. Secondly, for high-level features, an improved Simple Pyramid Pooling Module (SPPM) is used to enhance feature extraction; Finally, the improved Unified Attention Fusion Module (UAFM) is used to fuse the low-level feature and the high-level feature. The Li-DeepLabV3+ model not only ensures lightweight but also takes into account the accuracy of polyp segmentation. The remainder of this paper is structured as follows: In Section 2, we introduce the principle of the polyp segmentation algorithm based on Li-DeepLabV3+. In Section 3, we provide the experimental results, and then compare and study the performance of Li-DeepLabV3+ and other segmentation algorithms. Section 4 is the conclusion and discusses future work.

## Principle of polyp segmentation algorithm based on Li-DeeplabV3+

**Figure 1:**
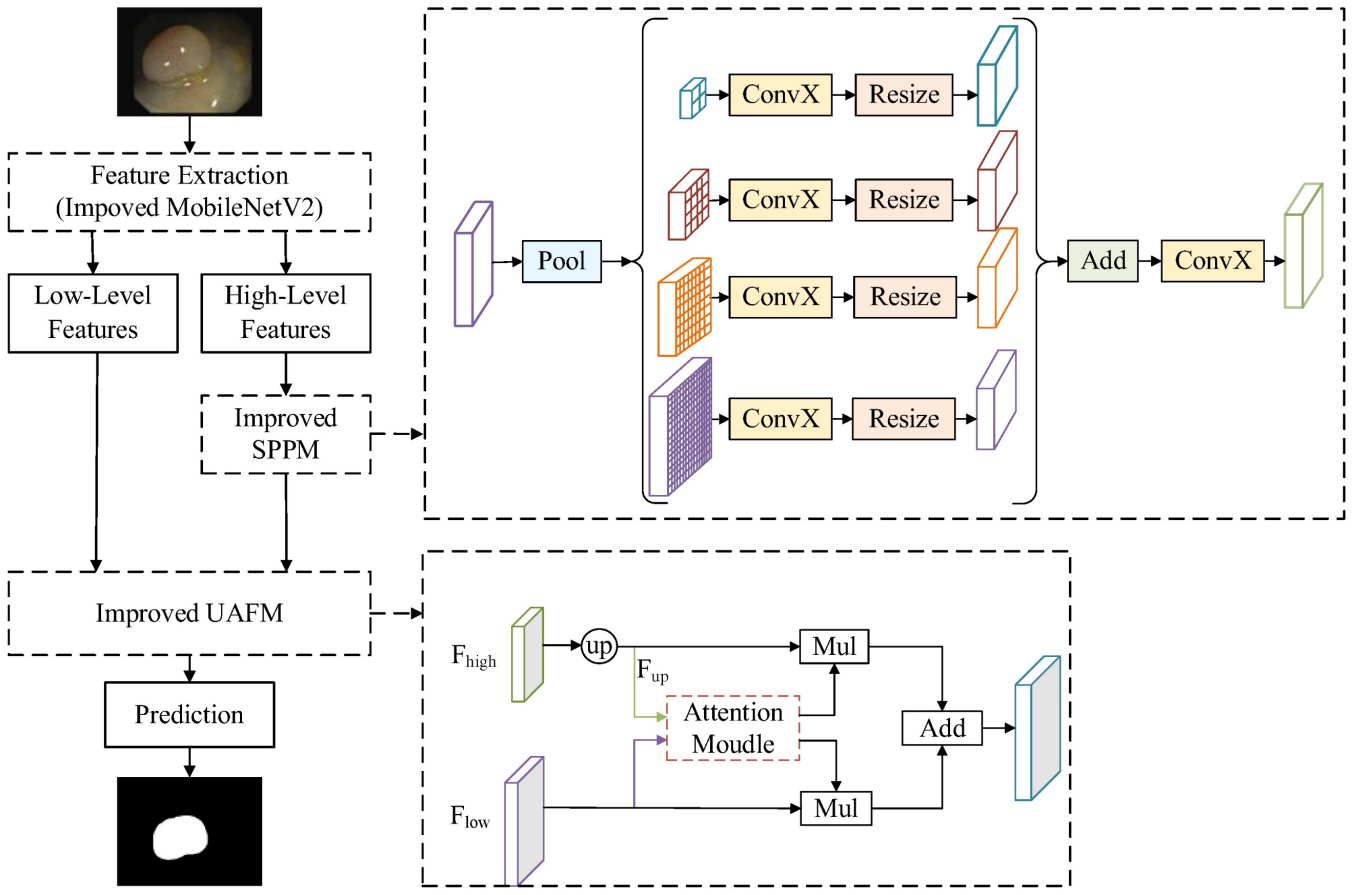
Li-DeepLabV3+ model structure diagram.

DeeplabV3+ 16 adopts an encoder-decoder structure to achieve segmentation. The encoder module extracts the features of the input image through the Xception 17 network, and the extracted high-level features are input to the Atrous Spatial Pyramid Pooling (ASPP) module to enhance feature extraction. The decoder module fuses the high-level feature and low-level feature after 4 times up-sampling, and then restores the original size of the image, thus completing the image segmentation. Polyp segmentation has high requirements in clinics, which needs to balance the accuracy and lightweight of the model. Therefore, a lightweight polyp segmentation algorithm Li-DeeplabV3+ is proposed, and its model structure diagram is shown in Fig. 1.

As shown in Fig. 1, the endoscope image first enters the optimized MobileNetV2 network for feature extraction to obtain a high-level feature and a low-level feature. The high-level feature is input into the Improved SPPM module to enhance feature extraction, and the Improved SPPM module improves the training efficiency of the model while reducing the model parameters; Secondly, the low-level features along with the processed high-level features are fed into the Improved UAFM module to enhance the feature representation; Finally, the feature map is input into the prediction module to realize the segmentation of polyp region.

### Improved MobileNetV2

**Figure 2:**
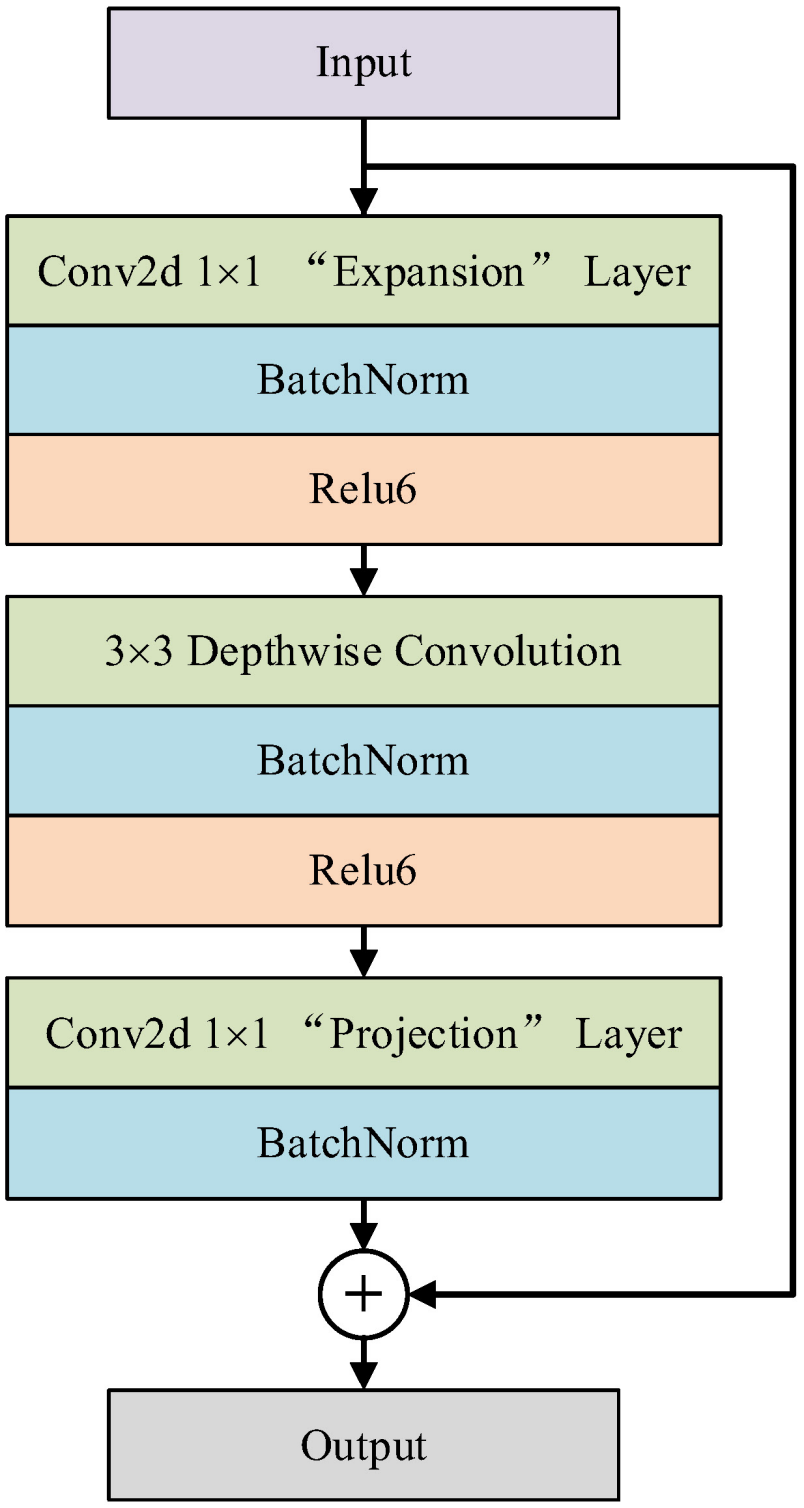
Inverse residual structure.

Because polyp segmentation requires high segmentation speed and accuracy in a computer-aided diagnosis system, the algorithm should try its best to ensure the recognition accuracy of global information when extracting image features, and at the same time, there are no redundant parameters and computation to ensure the efficiency of polyp segmentation. In this paper, the classic DeeplabV3+ network structure is improved by lightweight, and the backbone network is replaced by a pruned MobileNetV2 network instead of the Xception network. The whole MobileNetV2 18 network is mainly based on Inverted Residual Block, which can realize faster training and higher accuracy, and the model structure is easy to transplant and optimize. The inverted residual structure is shown in Fig. 2, which includes two parts: the left side is the trunk part. Firstly, 1 × 1 convolution is used to increase the dimension, then 3 × 3 Depthwise Separable Convolution is used to extract features across feature points, and finally, 1 × 1 convolution is used to reduce the dimension; On the right is the residual part, and the input and output are implemented by adding operation.

Among them, depth-separable convolution uses different convolution checks and different input channels to realize space and channel separation, which greatly reduces the computation and memory size of the network model and improves the running speed. In addition, the Relu6 activation function is used in the lifting dimension and feature extraction part of the inverted residual network, and the maximum output of the Relu activation function is limited to 6, which reduces the model parameters and improves the numerical resolution, and enhances the stability and generalization of the model 19.

In the DeeplabV3+ network model, the MobileNetV2 network is used as the backbone network to realize feature extraction and multi-feature layer fusion. Therefore, in this paper, the MobileNetV2 network is pruned, the three-layer structure used to realize classification is discarded, and the original network parameters are modified to carry out only four times downsampling to ensure the image resolution and segmentation accuracy. Taking 512×512×3 images as input, the structure of the pruned MobileNetV2 network is shown in Table 1. where Operator represents different network module layers, t represents dimension promotion multiple, c represents Output number, n represents the number of times the modules of this layer are used, s represents convolution step size, and Output represents the Output characteristic map size of each module, that is, the input layer of the next module.

### Improved SPPM

**Figure 3:**
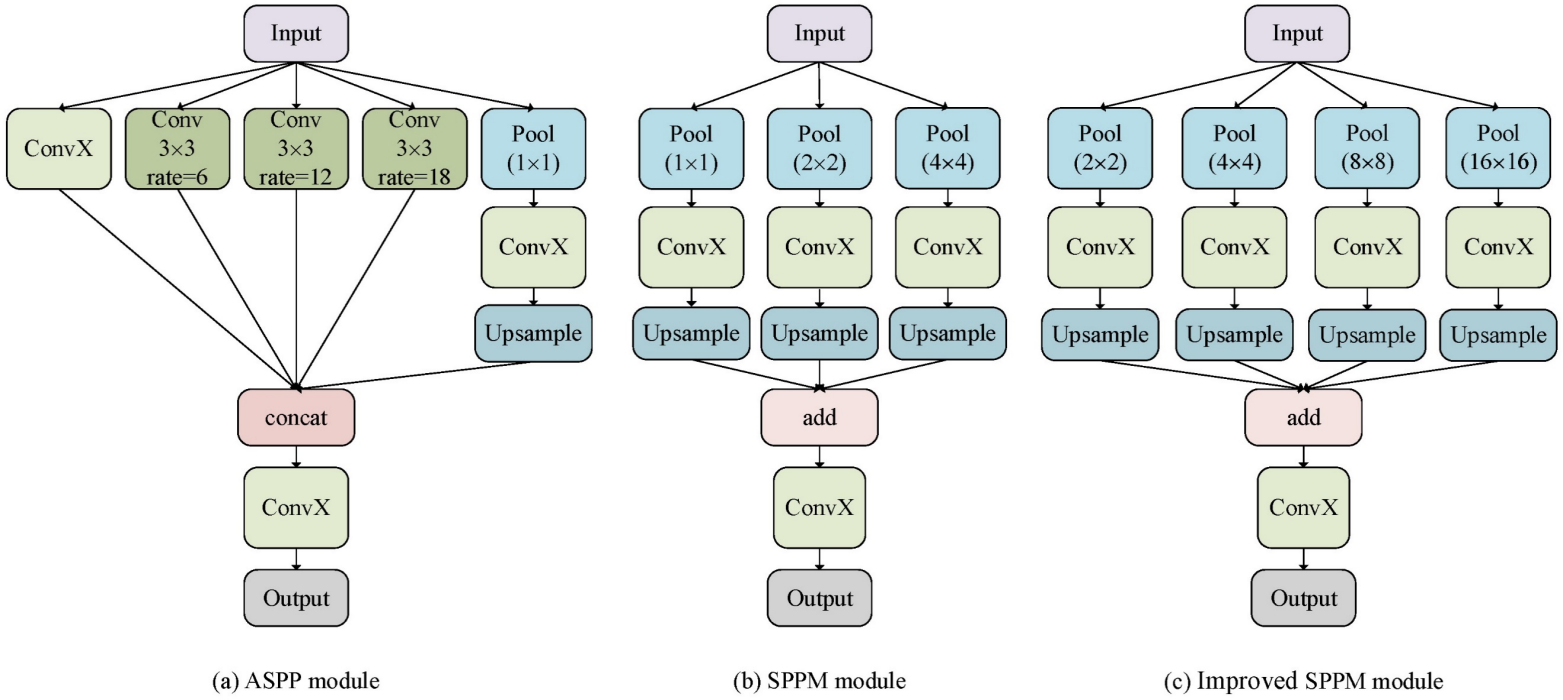
Improved SPPM module.

The ASPP module of the DeeplabV3+ model has a large amount of computation, so an improved SPPM is used to replace the original ASPP structure. The SPPM module uses adaptive pooling operation to collect context information and makes the global pooling operator output feature maps with sizes of 1 × 1, 2 × 2, and 4 × 4 respectively 20. To reduce the loss of image details when collecting context information, the original SPPM module is improved, and its global pooling operator outputs feature images with sizes of 2 × 2, 4 × 4, 8 × 8, and 16 × 16 respectively. Figure 3 shows the specific module structure diagrams of the ASPP, SPPM, and improved SPPM modules.

As shown in Fig. 3(c), The improved SPPM module first uses the pyramid pooling module to fuse the input features. The pyramid pooling module has four global averaging pooling operations to obtain feature maps of sizes 2 × 2, 4 × 4, 8 × 8, and 16 × 16, respectively. Then, the output features are followed by convolution and upsampling operations. For the convolution operation, the kernel size is 1 × 1 and the output channels are smaller than the input channels. Finally, the Add operation is performed on these upsampled features and the convolution operation is applied to generate the refined features. The above process can be expressed as equations (1)-(2).


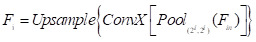

(1)


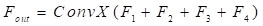

(2)

Where, ConvX denotes convolution, batch norm, and relu operations. The bin sizes of global average pooling are 2 × 2, 4 × 4, 8 × 8 and 16 × 16 respectively.

Compared with the original SPPM module, the improved SPPM module results in less loss of image details, thus achieving better segmentation results. Compared with the ASPP module, the improved SPPM module reduces the number of parameters, which better satisfies the real-time needs of the model.

### Improved UAFM

**Figure 4:**
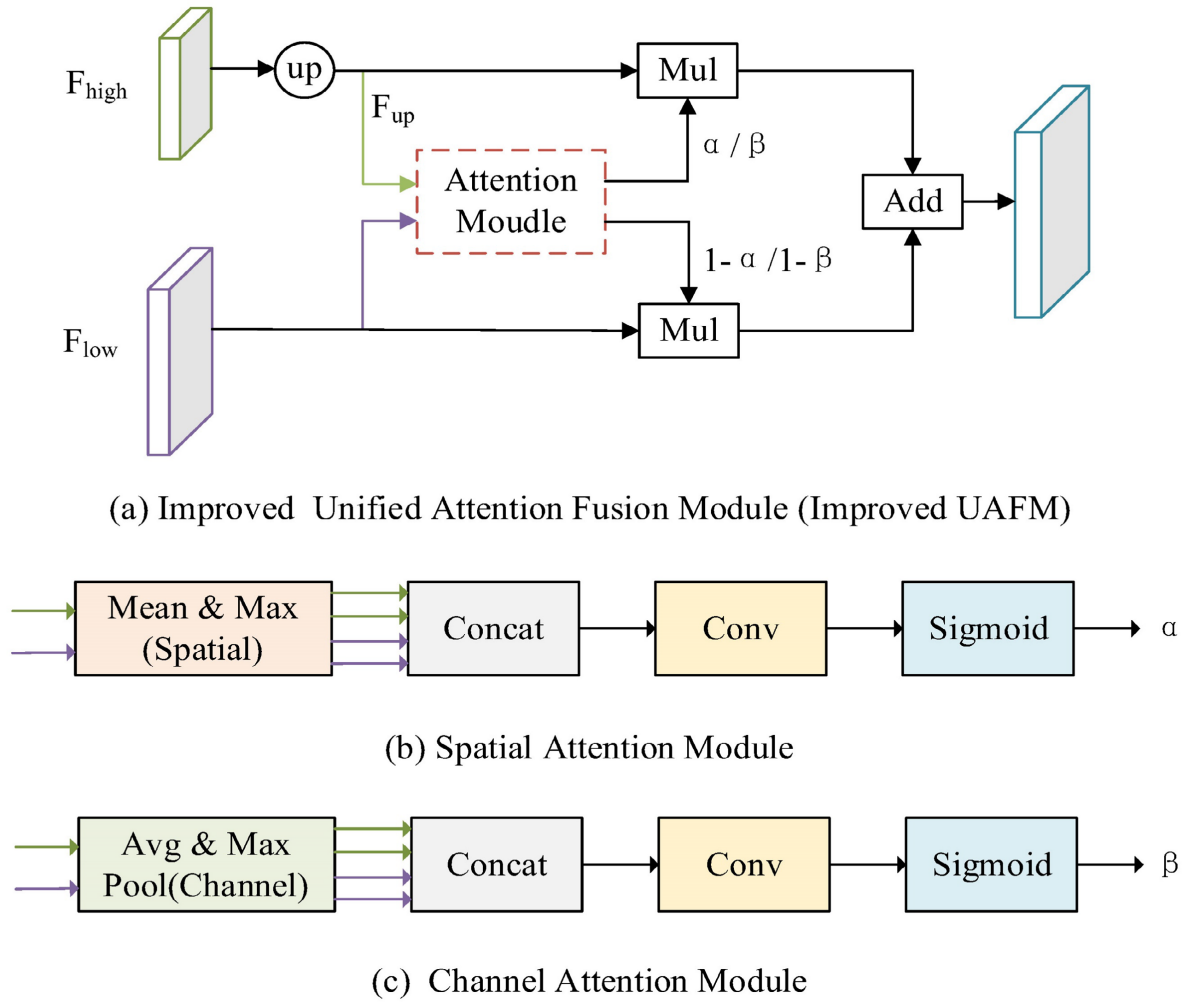
Improved UAFM module.

To enhance the feature representation and further reduce the parameters of the model, the Improved UAFM module is used to fuse high-level and low-level features. Traditional UAFM modules generate weights by using spatial or channel attention alone and then fuse input features with weights 20. To fuse features more efficiently, the traditional UAFM module is improved, and at the same time, the spatial and channel attention is used to further enhance the feature representation, to achieve clearer segmentation of polyp boundaries. The specific feature fusion module is shown in Fig. 4.

In Fig. 4 (a), F_high_ is a high-level semantic feature, F_low_ is a low-level semantic feature, and Attention Moulde in Improved UAFM has two modules: spatial attention module and channel attention module, which are represented by Fig. 4 (b) and Fig. 4 (c) respectively. Firstly, bilinear interpolation is used for the input F_high_ to make it the same size as F_low_, and it is expressed by F_up_; Secondly, the spatial attention module and the channel attention module generate weights α and β by taking F_up_ and F_low_ as inputs respectively, and then carry out Mul operation with F_up_ and F_low_ and then carry out Add operation to obtain a feature map, and use weight β to obtain another feature map in the same way; Finally, Mul operation is performed on the two feature graphs. The above process can be expressed as equations (3)-(6).


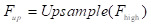

(3)


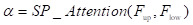

(4)


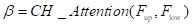

(5)


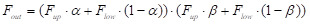

(6)

where SP_Attention indicates that the spatial attention module uses the relationship in space to generate weights, which represent the importance of each pixel in the input feature, and its formula is as shown in (7); CH_Attention means that the channel attention module uses the relationship between channels to generate weights, which indicate the importance of each channel in the input features, and its formula is shown in (8).


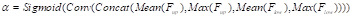

(7)


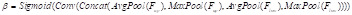

(8)

### Loss function

To alleviate the effect of uneven distribution of polyps and background pixels on model training, a mixed loss function of Focal Loss and Dice Loss is used to optimize the model 21. The formulas are shown in (9)-(12).


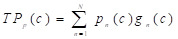

(9)


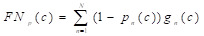

(10)


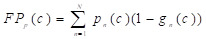

(11)


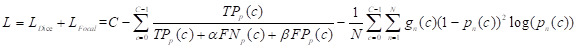

(12)

where 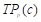, 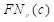,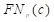, and 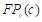 are the true positives, false negatives, and false positives of the categories using predicted probabilities, respectively; 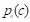 denotes the predicted probability of pixel n for the category 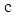; 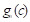 is the ground truth for voxel 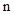 being class 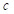; 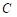 is the total number of segmentation categories plus background, and in this paper, it is necessary to segment highlights and background, so 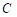 is equal to 2; 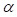 and 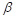 are trade-offs for penalties for false negatives and false positives, set to 0.5 in the experiment; 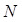 is the total number of pixels in the endoscopic image.

## Experimental analysis

In this paper, we use CVC-ClinicDB 4 and Kvasir-SEG 22 to train and validate the models. CVC-ClinicDB contains 612 images, both with a resolution of 384×288 pixels; Kvasir-SEG contains 1000 polyp images and corresponding labels, with resolutions ranging from 332×487 to 1920×1072 pixels. For CVC-ClinicDB, 80% of these images and masks were used for training, the remaining 10% for validation, and 10% for testing. For Kvasir-SEG, following the official split, 880 images and their masks were used to train the model, while the remaining 120 images and their masks were used for testing.

Our proposed Li-DeeplabV3+ is implemented in Python 3.8 using Pytorch framework on a workstation with Windows 11 system, NVIDIA GeForce RTX 3060 GPU, 11 GB RAM, and Intel Core i7 with 16 logical processors. The training of the model is combined with the transfer learning idea by pre-training weights from the VOC dataset 23. The number of training iterations for the model is set to 100, using the AdamW optimizer its initial learning rate is set to 0.0005 and the learning rate decreases using cosine annealing.

Experiments were conducted to evaluate the segmentation results using performance metrics that are widely used in the field of medical image segmentation, which was measured using five metrics: Intersection over Union (IoU), Dice coefficient (Dice), Precision (Pre), Recall (Re), and Accuracy (Acc). The above metrics are calculated as follows:


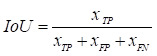
,
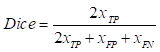
,
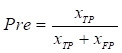



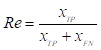
,
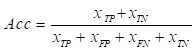


Where 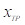 is the number of true positive pixels; 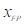 is the number of false positive pixels; 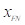 is the number of false negative pixels.

### The influence of transfer learning on the improved model

**Figure 5:**
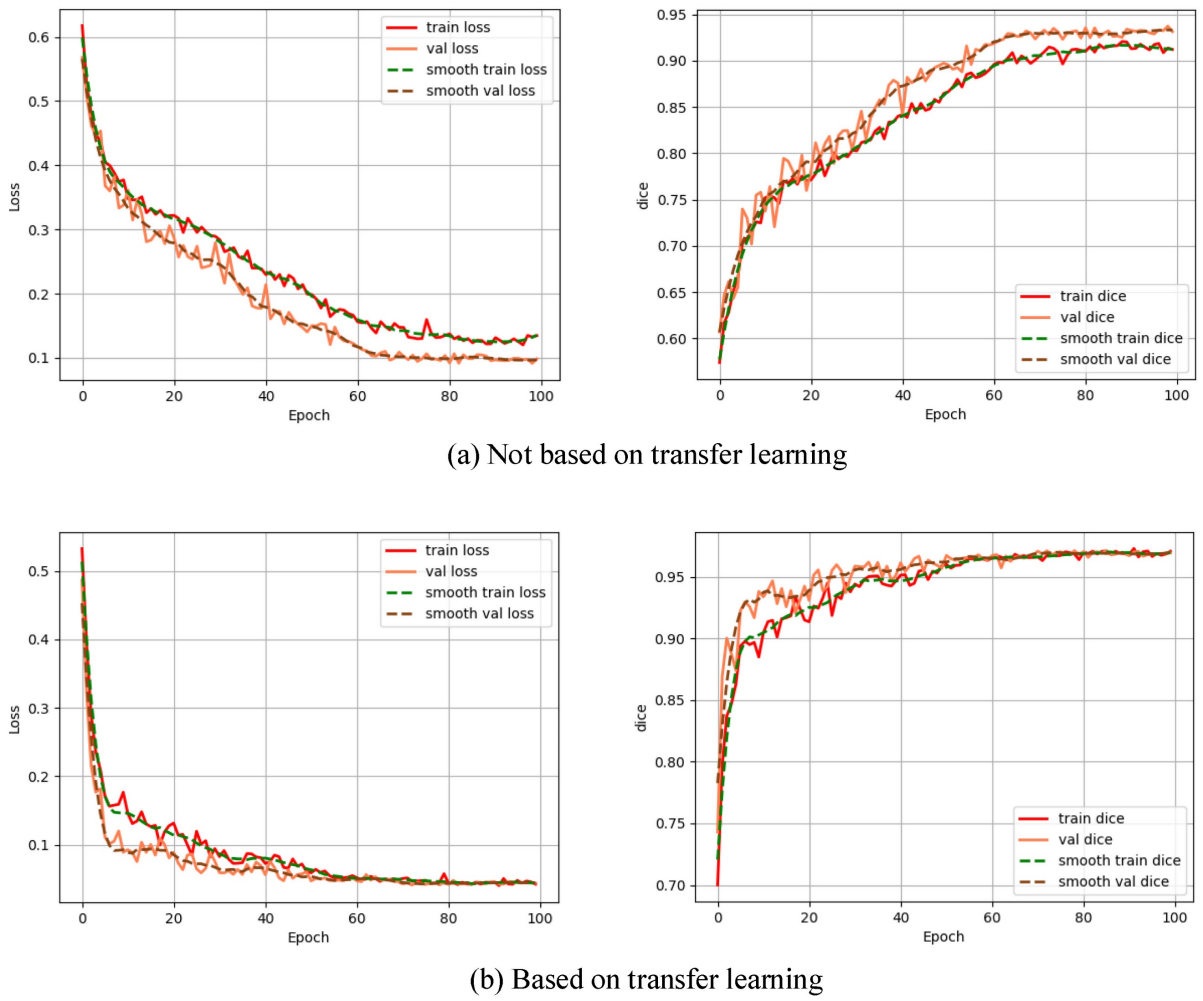
Impact of transfer learning on the improved model.

To explore the impact of transfer learning on the improved model, the model was trained by not based on transfer learning and based on transfer learning, respectively, and the Dice curves as well as the loss function curves of the different training methods are shown in Fig. 5.

From Fig. 5, we can see that non-based transfer learning needs to be trained about 80 times before the loss function converges to a stable value, whereas the loss function stabilizes at 60 times of training when the model is trained based on transfer learning. In addition, the training method based on transfer learning is more stable in terms of loss change during the training process than not based on transfer learning, which indicates that the training method based on transfer learning can learn the features of the image more quickly. Each evaluation index is shown in Table 2.

From Table 2, it can be seen that each evaluation index based on transfer learning has a large improvement compared with not based on transfer learning, which further illustrates the effectiveness of the transfer learning based on this improved model, which not only improves the training efficiency but also greatly improves the polyp segmentation effect.

### Influence of different loss functions on experimental results in improved model

**Figure 6:**
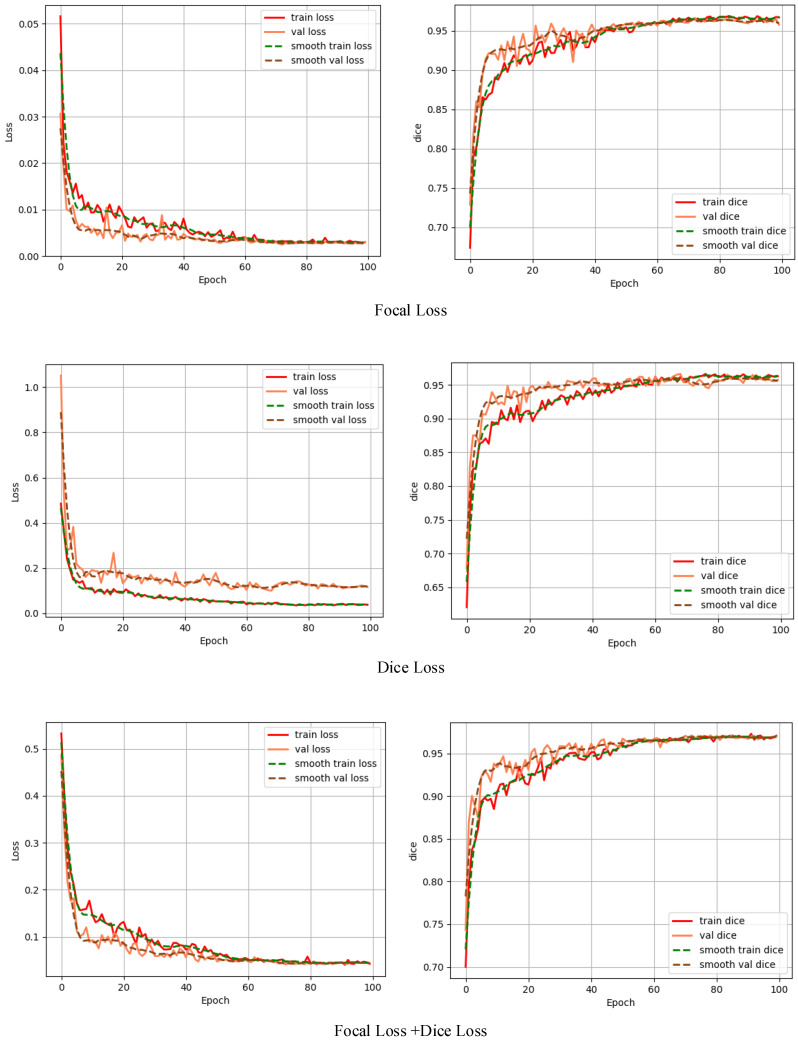
Loss function curve and Dice curve.

To study the effect of different loss functions on the polyp segmentation performance of the Li-DeepLabV3+ network, the comparative experiments of Dice loss 24, Focal loss 25, and the mixed loss function of Dice loss and Focal loss were carried out respectively. Each loss function curve and Dice curve are shown in Fig. 6.

From Fig. 6, because the actual situation of the samples in this data set is that the number of polyps and background pixels is unbalanced when using Focus loss alone to train this model, the model can learn poorly classified voxels, but the dice value fluctuates greatly during the training process. When using Dice loss alone for network training, it can focus on polyp region mining, but because of the small area of polyp region in the data set, the segmentation result is not very superior; The mixed loss function of Dice loss and Focal loss combines the characteristics of the two loss functions. In the process of network training, stable and targeted optimization is adopted for samples that are difficult to learn, thus alleviating the problems of sample imbalance and small target samples. Each evaluation index is shown in Table 3.

It can be seen from Table 3 that the evaluation indexes obtained by the mixed loss function are higher than those of the two loss functions used alone, and IoU is 1.21% and 2.49% higher than Focal loss and Dice loss, respectively, indicating that the mixed loss function is more suitable for solving the imbalance between positive and negative samples and containing small target samples, and can obtain better segmentation performance.

### Ablation experiment

**Figure 7:**
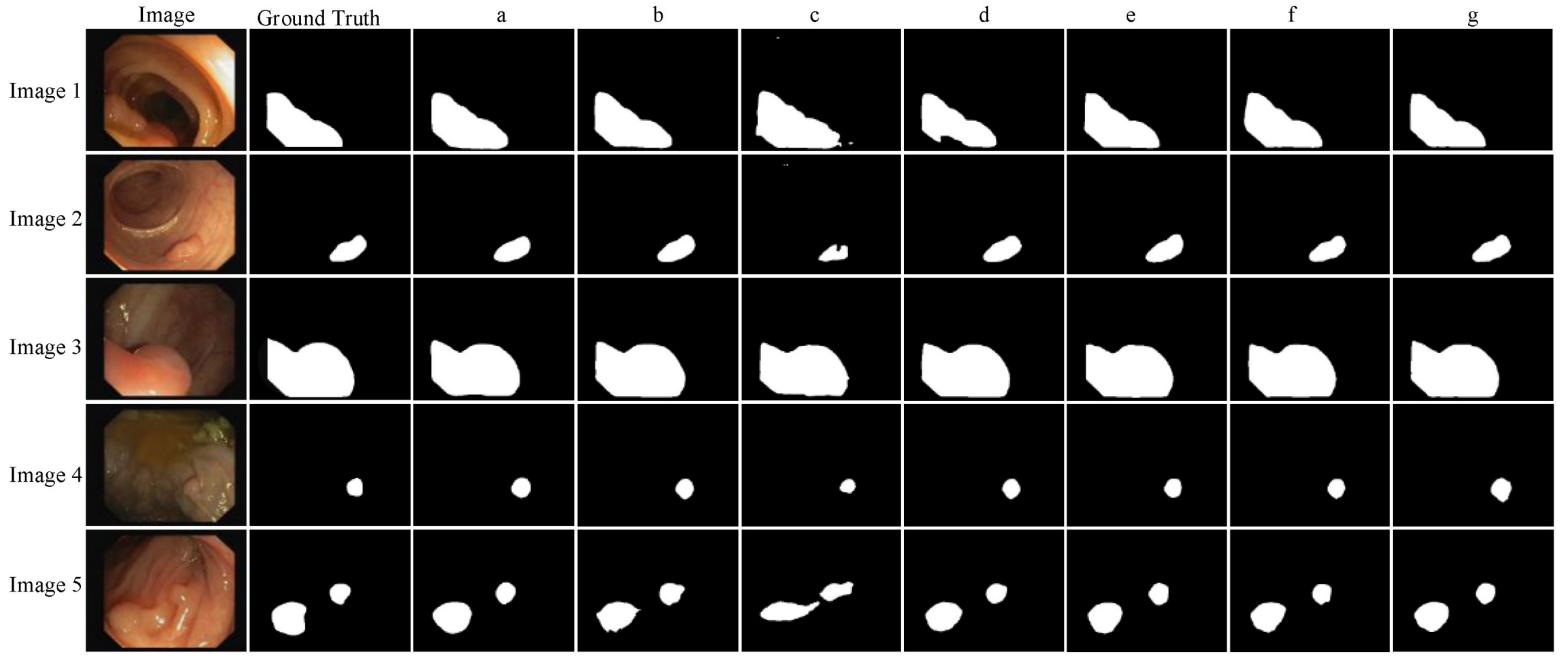
Ablation experiment.

The proposed model is applied to the CVC-ClinicDB data set under the same experimental conditions. The effects of the backbone network feature enhancement module and feature fusion module on the segmentation performance and speed are mainly observed. The performance evaluation index results are shown in Table 4 (where Params represents the total number of parameters to be trained in the network model, the unit M represents Million, and FPS represents the number of frames transmitted per second), and the visualization results of segmentation performance are shown in Fig. 7.

It can be seen from Table 4 that compared with the backbone network Xception, when the backbone network uses mobilenetV2, although the IoU and Dice parameters of the network are slightly lower, the model training parameters are nearly ten times less, and the FPS is also increased by about two times. Because of the need for polyp segmentation for model segmentation speed, the backbone network mobilenetV2 is more suitable. It can be seen from Fig. 7 that the edge of Image 5 polyp segmentation of method b appears burrs, and the segmentation is inaccurate, which needs further improvement. To further reduce the number of parameters and improve the segmentation effect of the network, this paper improves the feature enhancement and feature fusion module of the model.

For the feature enhancement module, when the original feature enhancement module ASPP is replaced by the SPPM module, the parameters of the model are reduced by 1.87M again, and the FPS reaches 89.92, but the segmentation effect of the model is poor. Therefore, the SPPM module is improved. Although the FPS using the Improved SPPM module is reduced by 2.28, the parameters of the model are only increased by 0.082 M, while the IoU and Dice parameters are increased by 8.56% and 5.21% respectively. It can also be seen in Fig. 7 that the segmentation effect of the d method is better than that of the c method, which shows the effectiveness of Improved SPPM.

For the feature fusion module, when the original feature fusion module is replaced by the UAFM module, the parameters of the model are reduced by 0.482 M, and the accuracy is improved. To further improve the segmentation effect, the UAFM module is improved. The IoU and Dice coefficient of the model are increased to 87.05% and 93.07% respectively with the same parameters, which proves the superiority of the Improved UAFM.

When Improved UAFM and Improved SPPM are used at the same time, the parameters of the improved model are reduced by 15.4 times compared with the original model, FPS is increased by two times, IoU, Dice coefficient and accuracy of the model reach 87.42%, 93.28%, and 98.80% respectively, and Recall and Precision reach an equilibrium state. The advantages of the method can also be reflected in Fig. 7, and the segmentation contour is closer to Ground Truth, so Li-DeeplabV3+ can meet the requirements of polyp segmentation speed and achieve better segmentation accuracy.

### Quantitative analysis

**Figure 8:**
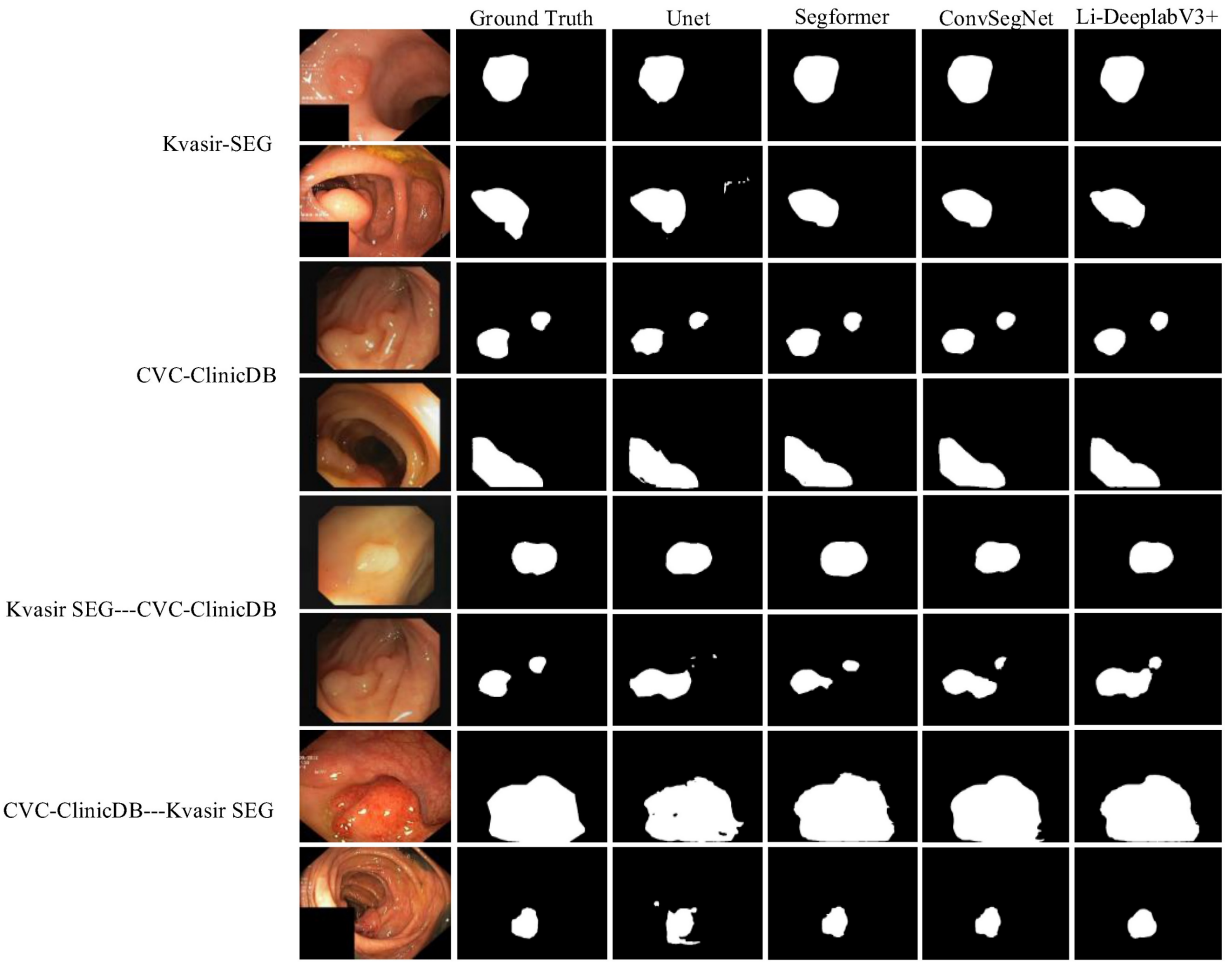
Quantitative analysis.

To test the superiority of the improved algorithm, Kvasir-SEG, and CVC-ClinicDB datasets are used to compare three segmentation algorithms, Unet 26, SegFormer 27, and ConvSegNet 11. At the same time, two cross-dataset experiments are carried out to explore the generalization ability of the improved model. The image size is adjusted to 256 × 256, and the training is carried out under the same conditions. The visualization result of segmentation performance is shown in Fig. 8.

It can be seen from Fig. 8 that the segmentation results of the Li-DeepLabV3+ model on Kvasir-SEG and CVC-ClinicDB data sets are closer to Ground Truth than other models, which can suppress similar background areas and noises more thoroughly, and have stronger robustness to the shape and size changes of target areas. For cross-datasets, the improved model is more delicate and more generalized than other models. Specific segmentation evaluation indexes are shown in Table 5.

It can be seen from Table 5 that for the Kvasir-SEG data set, IoU, Dice coefficient, recall, and accuracy of the Li-DeepLabV3+ model are the best results, reaching 79.88%, 88.82%, 88.42%, and 96.56% respectively. The recall rate of the Li-DeepLabV3+ model is 1.81% lower than that of the ConvSegNet model, but its accuracy rate is 88.42%, which indicates that the possibility of false detection of this model is less. For the CVC-ClinicDB data set, the evaluation indexes of this model are better than all benchmark models, and the accuracy and recall are balanced, which has strong clinical application potential. IoU is 2.75%, 9.84%, and 3.97% higher than Unet, SegFormer, and ConvSegNet respectively, which reflects the superiority of the improved algorithm.

For the cross-dataset experiment from Kvasir-SEG to CVC-ClinicDB dataset, compared with the most competitive ConvSegNet model, the accuracy of IoU, Dice coefficient, Precision, and Recall of the improved model is 2.6%, 5.13%, 4.2%, 0.79% and 0.01% higher, respectively. From the segmentation results in Fig. 8, it can be seen that the possibility of missing detection is smaller than in other models. After training the model on CVC-ClinicDB and testing it on Kvasir-SEG, the accuracy and Dice of the improved model reached 94.79% and 81.36%, respectively. The recall is 8.35% lower than that of ConvSegNet, but the precision rate is 14.56% higher than that of ConvSegNet, which shows that the Li-DeepLabV3+ model has a certain generalization ability.

**Figure 9:**
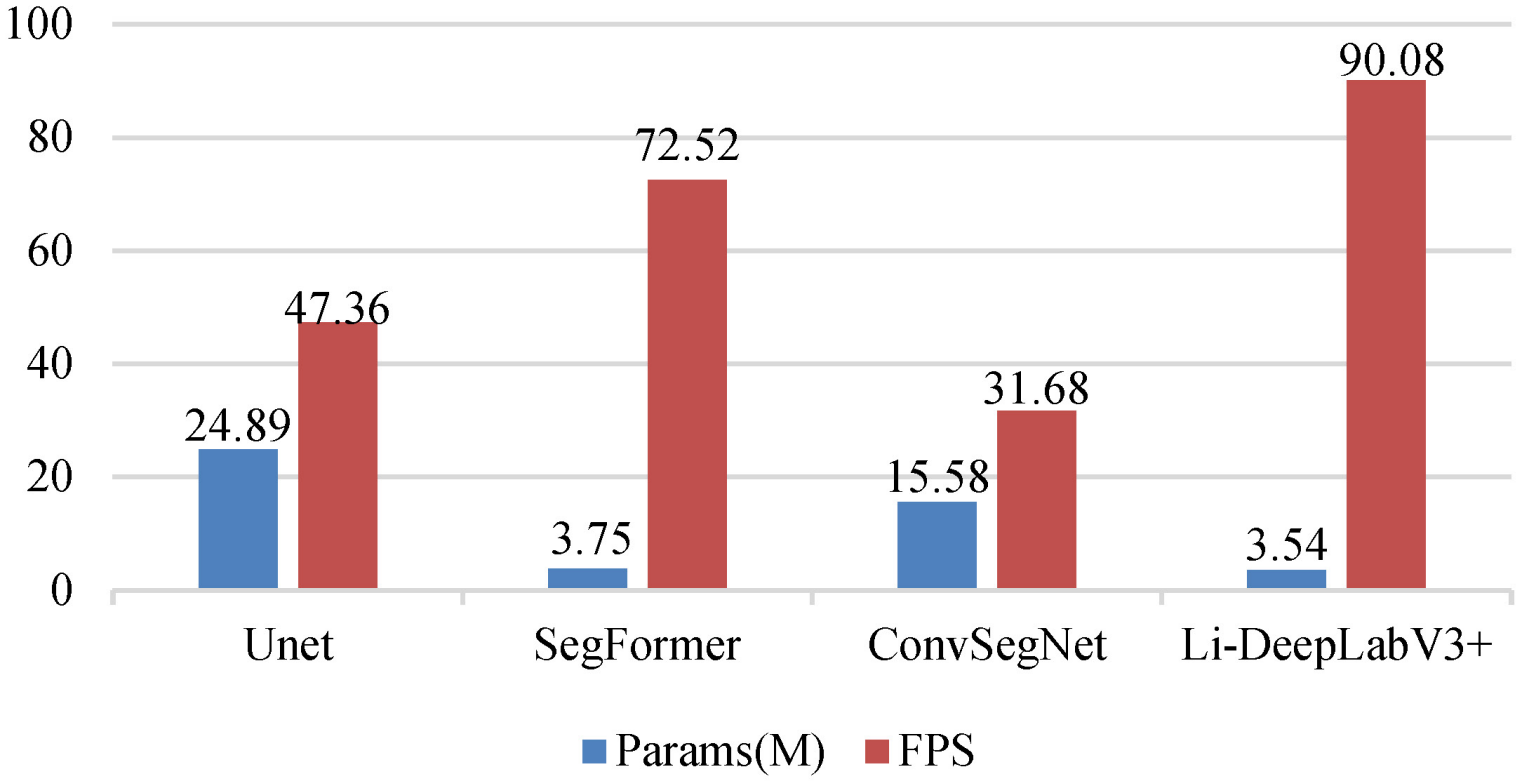
Comparison of Li-DeeplabV3+ and benchmark model parameters and FPS.

To explore the lightweight and real-time nature of the model, the number of parameters as well as the FPS of the above models are compared under the same experimental conditions, and the results are shown in Fig. 9.

As can be seen from Fig. 9, the Li-DeeplabV3+ model has the fastest segmentation speed with the least parameters, and its parameters are 21.35 M, 0.21 M, and 12.04 M less than those of UNet, SegFormer, and ConvSegNet, respectively. At the same time, the FPS of the model is superior to all benchmark models, among which, compared with the most competitive SegFormer model, the FPS of Li-DeeplabV3+ is improved by 17.56. This further proves that the proposed method takes into account the lightweight and real-time performance of the polyp segmentation model.

### Clinical experiments

**Figure 10:**
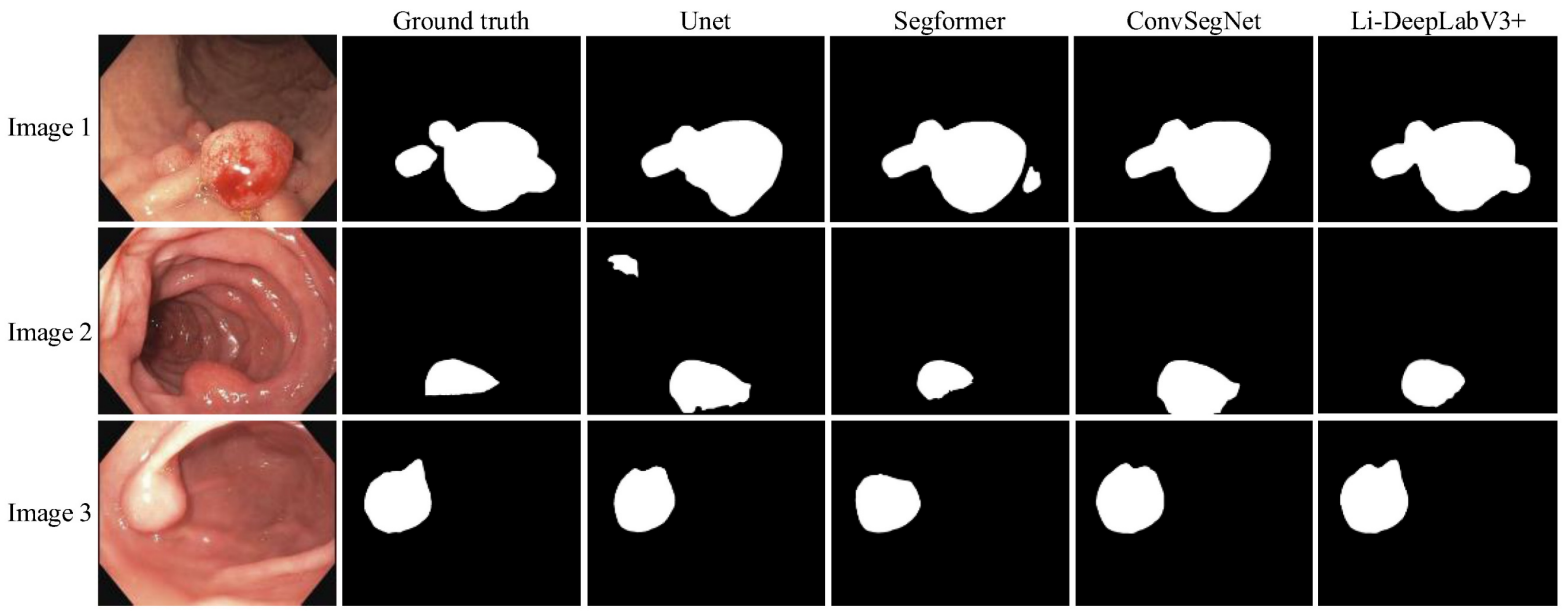
Segmentation effect on the self-made datasets.

To validate the Li-DeepLabV3+ model for clinical segmentation, we performed experiments on a self-made clinical dataset using pre-trained weights which were trained on the Kvasir-SEG dataset. The segmentation results of different algorithms are shown in Fig. 10, where Ground truth is the result of manual segmentation by relevant experts.

From Fig. 10, it can be seen that the segmentation results of our proposed model Li-DeepLabV3+ are closer to Ground truth relative to other models. lower miss and false detection rates, and the lesion area can be segmented more accurately. The specific segmentation evaluation indexes are shown in Table 6.

It can be seen from Table 6 that compared with other models, the Li-DeepLabV3 + model is at the best level of IoU, Dice, precision, and accuracy, and at the same time, the accuracy and recall rate are in a balanced state. This proves the superiority of the Li-DeepLabV3 + model and its effectiveness and feasibility in clinical practice.

## Conclusion

An end-to-end lightweight polyp segmentation network model Li-DeepLabV3+ is proposed in this paper. Firstly, the backbone network of the original DeeplabV3+ model is replaced by the optimized MobileNetV2 network to reduce the complexity of the model; Secondly, the Improved SPPM is adopted in the feature extraction module to reduce the model parameters and improve the training efficiency of the model; Finally, Improved UAFM is used to fuse low-level features and high-level features to enhance the feature representation of the model. The parameters of the Li-DeeplabV3+ model are reduced by 15 times compared with the original model, and IoU and Dice of 79.88%, 88.82%, and 87.42%, 93.28% are obtained on Kvasir SEG and CVC-ClinicDB respectively. Compared with the benchmark model in the experiment, the Li-DeeplabV3+ model has both accuracy and real-time performance of polyp segmentation and has a certain generalization ability. Although the parameters of the Li-DeepLabV3+ model have been significantly reduced, compared with the latest model, its missing detection rate can not be ignored. For future work, we plan to explore how to reduce the missing detection rate of polyp region segmentation while ensuring the lightweight of the model.

## Figures and Tables

**Table 1:** MobilenetV2 network structure after pruning.

Operator	t	c	n	s	Output
Conv2d 3×3	-	32	1	2	256×256×32
Bottleneck	1	16	1	1	256×256×16
Bottleneck	6	24	2	2	128×128×24
Bottleneck	6	32	3	2	64×64×32
Bottleneck	6	64	4	2	32×32×64
Bottleneck	6	96	3	1	32×32×96
Bottleneck	6	160	3	1	32×32×160
Bottleneck	6	320	1	1	32×32×320

**Table 2:** Impact of transfer learning on the improved model.

Methods	IoU(%)	Dice(%)	Pre(%)	Re(%)	Acc(%)
Not based on transfer learning	82.27	90.27	90.09	90.45	98.26
Based on transfer learning	**87.42**	**93.28**	**93.05**	**93.54**	**98.80**

**Table 3:** Performance comparison of different loss functions on the improved model.

Methods	IoU(%)	Dice(%)	Pre(%)	Re(%)	Acc(%)
Focal 24	86.21	92.59	92.56	92.63	98.68
Dice 25	84.93	91.85	91.37	92.34	98.54
Focal+dice 21	**87.42**	**93.28**	**93.05**	**93.54**	**98.80**

**Table 4:** Ablation experiment.

Methods		IoU(%)	Dice(%)	Pre(%)	Re(%)	Acc(%)	Params(M)	FPS
a	Xception+ASPP^16^	86.64	92.84	92.17	93.52	98.71	54.71	44.82
b	mobilenetV2+ASPP	85.97	92.46	92.02	92.89	98.65	5.81	85.93
c	mobilenetV2+SPPM	77.05	87.04	85.48	88.66	97.64	3.94	89.92
d	mobilenetV2+ Improved SPPM	85.61	92.25	90.98	93.54	98.63	4.03	87.64
e	mobilenetV2 +ASPP+UAFM	86.81	92.91	**93.15**	92.73	98.74	5.33	87.37
f	mobilenetV2+ASPP+Improved UAFM	87.05	93.07	92.82	93.33	98.76	5.33	86.58
g	Li-DeeplabV3+	**87.42**	**93.28**	93.05	93.54	**98.80**	**3.54**	**90.08**

**Table 5:** Quantitative analysis.

Method	IoU(%)	Dice(%)	Pre(%)	Re(%)	Acc(%)
Dataset: Kvasir-SEG					
Unet 26	73.84	84.95	88.17	81.11	95.38
SegFormer 27	76.94	86.97	86.07	87.89	96.51
ConvSegNet 11	79.44	86.30	86.23	**91.03**	95.72
Li-DeepLabV3+ (ours)	**79.88**	**88.82**	**88.42**	89.22	**96.56**
**Dataset: CVC-ClinicDB**					
Unet 26	84.67	91.70	94.50	89.06	98.74
SegFormer 27	77.58	87.37	89.55	85.30	97.80
ConvSegNet 11	83.45	90.27	89.64	92.58	98.79
Li-DeepLabV3+ (ours)	**87.42**	**93.28**	**93.05**	**93.54**	**98.80**
**Training dataset: Kvasir-SEG----Test dataset: CVC-ClinicDB**					
Unet 26	61.18	75.92	80.08	72.16	96.44
SegFormer 27	67.69	80.73	83.38	78.25	96.66
ConvSegNet 11	68.35	77.87	79.64	81.41	96.98
Li-DeepLabV3+ (ours)	**70.95**	**83.00**	**83.84**	**82.20**	**96.99**
**Training dataset: CVC-ClinicDB----Test dataset: Kvasir-SEG**					
Unet 26	66.66	79.99	81.52	78.53	94.38
SegFormer 27	67.20	80.38	82.13	78.71	94.50
ConvSegNet 11	61.63	71.55	69.18	**87.89**	90.13
Li-DeepLabV3+ (ours)	**68.58**	**81.36**	**83.74**	79.35	**94.79**

**Table 6:** Segmentation effects on self-made datasets.

Methods	IoU(%)	Dice(%)	Pre(%)	Re(%)	Acc(%)
Unet^26^	82.74	90.56	85.58	96.15	98.03
Segformer^27^	81.05	89.53	92.16	87.05	98.00
ConvSegNet^11^	78.07	86.94	79.93	**97.42**	97.48
Li-DeepLabV3+(ours)	**87.91**	**93.56**	**93.27**	93.86	**98.74**
